# Predicting Progression from Normal to MCI and from MCI to AD Using Clinical Variables in the National Alzheimer’s Coordinating Center Uniform Data Set Version 3: Application of Machine Learning Models and a Probability Calculator

**DOI:** 10.14283/jpad.2023.10

**Published:** 2023

**Authors:** Y. Pang, W. Kukull, M. Sano, R.L. Albin, C. Shen, J. Zhou, H.H. Dodge

**Affiliations:** 1. Department of Computer Science and Engineering, Michigan State University, East Lansing, Michigan, USA;; 2. National Alzheimer’s Coordinating Center, University of Washington, Seattle, Washington, USA;; 3. Department of Psychiatry, Alzheimer Disease Research Center, Icahn School of Medicine at Mount Sinai, New York, New York, USA;; 4. Department of Neurology, Michigan Alzheimer’s Disease Center, University of Michigan, Ann Arbor, Michigan, USA;; 5. Neurology Service & GRECC, VAAAHS, Ann Arbor, MI, USA;; 6. Biogen, Inc, Cambridge, Massachusetts, USA;; 7. Department of Neurology, Layton Aging and Alzheimer’s Disease Center, Oregon Health & Science University, Portland, Oregon, USA;; 8. Department of Neurology, Massachusetts General Hospital, Harvard Medical School, Boston, Massachusetts, USA

**Keywords:** National Alzheimer’s Coordinating Center (NACC), Alzheimer’s disease, MCI, dementia, MCI conversion, AD, PET amyloid, machine learning

## Abstract

Clinical trials are increasingly focused on pre-manifest and early Alzheimer’s disease (AD). Accurately predicting clinical progressions from normal to MCI or from MCI to dementia/AD versus non-progression is challenging. Accurate identification of symptomatic progressors is important to avoid unnecessary treatment and improve trial efficiency. Due to large inter-individual variability, biomarker positivity and comorbidity information are often insufficient to identify those destined to have symptomatic progressions. Using only clinical variables, we aimed to predict clinical progressions, estimating probabilities of progressions with a small set of variables selected by machine learning approaches. This work updates our previous work that was applied to the National Alzheimer’s Coordinating Center (NACC) Uniform Data Set Version 2 (V2), by using the most recent version (V3) with additional analyses. We generated a user-friendly conversion probability calculator which can be used for effectively pre-screening trial participants.

## Introduction

Clinical trials are increasingly focused on treating Alzheimer‘s disease (AD) during early, asymptomatic, or pre-dementia stages. Differentiating those likely to progress in clinical diagnosis from normal to MCI or from MCI to dementia/AD from those unlikely to progress is challenging. Recruiting non-progressors into trials leads to reduced statistical power for detecting efficacy (1–3). As biomarkers are not necessarily correlated with clinical outcomes, using biomarkers alone as screening tools might not be optimal for trial enrichments, may be costly, and is susceptible to sample selection bias because of their invasive nature. The importance of accurate identification of progressors is expected to increase because of the recent FDA approval of aducanumab for MCI and mild dementia due to Alzheimer disease. The Centers for Medicare and Medicaid Services (CMS) made a decision to cover treatment costs with this monoclonal antibody only for Medicare beneficiaries enrolled in approved studies (https://www.cms.gov/medicare-coverage-database/view/ncacal-decision-memo.aspx?proposed=N&ncaid=305). Since this agent has a relatively high risk of amyloid-related imaging abnormalities (ARIA) (4), accurately predicting and enrolling those at higher risk of clinical progression into trials is critical for avoiding unnecessary treatments.

In the United States, National Institute of Health (NIH)-funded Alzheimer’s Disease Centers are required to use an uniform assessment approach (Uniform Data Set [UDS]) and upload this data to the National Alzheimer’s Coordinating Center (NACC) (https://www.naccdata.org). We previously used clinical variables from NACC UDS Version 2 (V2) and a machine learning approach to identify a set of variables predicting normal to MCI conversions within 4 years (5). UDS Version 3 (V3) replaced V2 in 2015 (6, 7). In the current study, we updated our analyses by using V3 with additional analyses. These additional analyses include: 1) using shorter durations of follow-ups to predict conversions from MCI to clinical diagnosis of AD (2 years and 3 years) and 2) generating a user-friendly calculator which estimates the probability of progression by entering subject-specific values of the small set of selected variables. Since patients interested in enrolling in anti-amyloid and other drug trials are likely to be referred to Alzheimer’s Disease Centers (ADCs) for in-depth diagnosis, pragmatic trials can be proposed by including participants who visited ADCs. The current study aims to provide the probability of conversion in clinical diagnosis with high accuracy using the NACC UDS V3 data. The calculator may be useful for pre-screening potential trial participants with high probabilities of clinical progressions before pursuing higher-cost biomarker assessments, such as an assessment of amyloid burden by a positron emission tomography (PET). The calculator can also be used for study enrichment for other trials and to strategize patients’ follow-up plans.

## Methods

### Data

#### NACC data

The NACC at the University of Washington maintains a repository of the UDS collected from participants in all of the National Institute on Aging (NIA)-funded Alzheimer’s Disease Centers (ADC) in the United States. There are over 30 past and present ADCs. The UDS consists of data collection protocols administered systematically to participants enrolled in each ADC (8–16). Participants are recruited, enrolled, and followed on an annual basis, generating center-specific longitudinal cohorts. These participants include individuals with clinical syndromic diagnoses of normal cognition (NC), MCI or cognitive impairments not meeting clinical MCI criteria, and dementia of various etiologies, including AD. Each AD Center enrolls participants in a NACC research cohort according to center-specific priorities. In general, most participants come from clinician referrals, self-referral by patients or family members, active recruitment through community organizations, and volunteers who wish to contribute to research. Most centers also enroll volunteers with normal cognition. NACC participants are not an epidemiologically based sample of the U.S. population with or without dementia. They are best regarded as a referral-based or volunteer case series selected based on each center’s research focus. Consent is obtained at the individual ADCs, as approved by their Institutional Review Boards (IRBs). The UDS data includes demographics, medical history, medication use, physical and neurological exam findings, clinical ratings of dementia severity [Clinical Dementia Rating (CDR^®^) Dementia Staging Instrument] (17), and neuropsychological test scores. Systematic guidelines for clinical diagnosis are based on the most up-to-date published diagnostic research criteria (8, 18–20). Further information on the NACC database may be found at: https://www.alz.washington.edu. Current analyses use data downloaded on March 12, 2021.

### Analytical Approaches

We have two independent objectives in this study: MCI progression prediction and AD progression prediction. In the MCI progression prediction, we aimed to differentiate those who converted to clinical diagnosis of MCI (or AD without having a diagnosis of MCI) within 4 years from baseline normal cognition and those maintaining normal cognition for at least 4 years. For this prediction task, a cognitively normal subject converting to MCI at any time within the 4-year observation window is a positive case, and a cognitively normal subject remaining normal for at least 4 years is used as a negative case. Cognitively normal subjects remaining normal by the time of loss to follow-up before year 4 were excluded. We also examined the transition to a specific subtype of MCI, amnestic MCI (aMCI), again with a 4-year observation window. Due to the relatively small sample size of non-amnestic MCI subjects in this cohort, we did not examine the transition to non-amnestic MCI (naMCI). In predicting the progression to clinical diagnosis of Alzheimer’s disease (AD), we aimed to differentiate between those converting to AD from MCI and those maintaining MCI status, using two follow-up durations: observation windows of 2 years and 3 years. We used these durations because the majority of clinical trials recruiting MCI participants complete follow-ups within 36 months.

#### Data preparation

Considerable time and effort were devoted to making the data appropriate for the supervised modeling process. Baseline numerical patient characteristics are included and missing indicators that indicate reasons for missingness (e.g., 99, −4) were coded appropriately by creating a dummy variable that indicates each type of missingness if they are considered to contribute to the prediction. After data clean-up and preparation procedures and determination of cognitive status at each assessment time point (see Supplement for more information), we applied the models listed below.

#### Feature selection method & Classification method

We examined the sensitivity, specificity, and accuracy of predictions using Receiver Operating Characteristics Area Under Curve (AUC). In this study, classifiers included Support Vector Machine (SVM), Logistic Regression (LR), and Random Forests. We compared the performance of univariate feature selection methods, including Information Gain (InfoGain), chi-squared test (Chi2), Fisher Score, and Analysis of variance (ANOVA). Additionally, embedded methods, including LASSO and Decision Tree, which incorporate feature selection within the classifier construction, and determine the feature importance simultaneously (joint feature selection property), were also used.

Different numbers of clinical variables (henceforth we call “features”) were examined (2, 5, 10, 15, 20, 25, 30, 35, and 40) to compare model performance. The number of features is determined when AUC does not improve significantly after including additional features. In our pipeline of model performance evaluation, datasets were divided into test and training datasets. We examined each combination independently by 5-fold cross-validation on the training datasets to automatically select the proper combination of our final model according to average validation performance. The whole assessment process was repeated 10 times to compute the average performance across the 10 repetitions. The final model was tested on the test datasets and corresponding performance metrics were reported.

#### Diagnosis

Cognitive statuses assigned during annual visits included normal cognition, MCI, aMCI, and AD. This information was extracted from the NACC data and used to determine progressions (i.e., from normal to MCI and from MCI to AD). The variables used to determine the cognitive status including etiology are described in detail in Supplement A and Supplement Table 1.

## Results

Table 1 summarizes the sample sizes used for each prediction. The largest sample size (number of unique participants) was for examining predictors of MCI to AD transition with 2 years (MCI2AD_2) because we could use samples with only 2 years of follow-up. The smallest sample size was for examining predictors of normal cognition to aMCI. Those who progressed to MCI within 4 years were reduced from 408 to 183 once we limited MCI incidence to aMCI.

**Figure 1.**
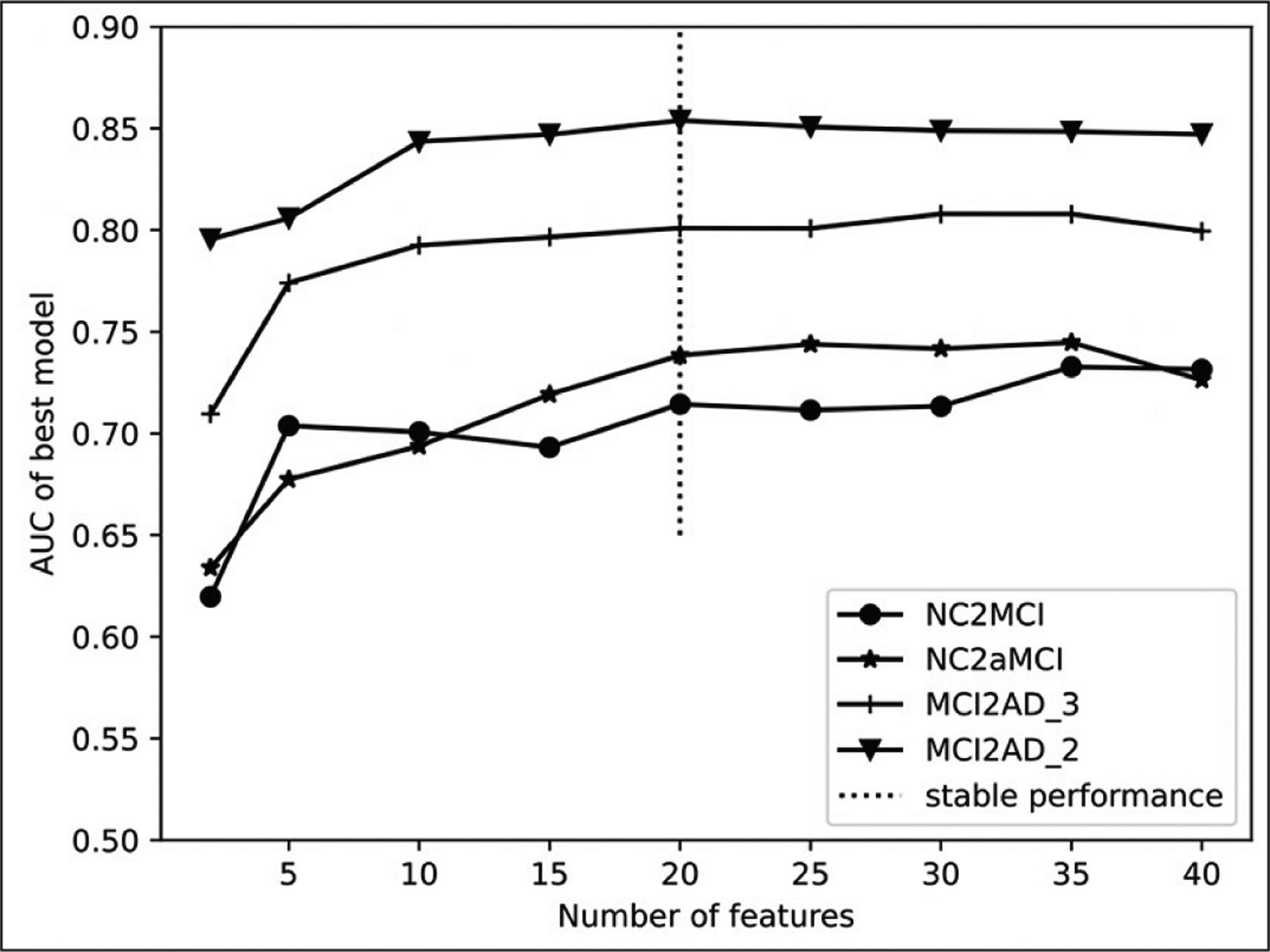
AUC of prediction models over different numbers of features

Figure 1 shows that 20 selected clinical variables were the best candidate parameter for our prediction model in the cross-validation process. That is, further adding features beyond 20 did not improve performance significantly. Therefore, the performance metrics were assessed using 20 clinical variables in the following model constructions.

### Prediction model performance

Tables 2 and 3 show results of the models’ test performances for each progression, including from normal cognition to MCI (NC2MCI) within 4 years, normal cognition to amnestic MCI within 4 years (NC2aMCI), MCI to AD within 2 years (MCI2AD_2) and MCI to AD within 3 years (MCI2AD_3). The AUC was lower for predicting MCI transition which was around 70%, compared with predicting AD transition, which was around 80%. For example, for the NC2aMCI transition, the combination using Random Forest as a classifier and ANOVA as a feature selection method reached the highest AUC with average 74.6% accuracy, 76.4% specificity, 71.6% sensitivity, and 74.0% AUC. For the MCI2AD_3 transition, the combination using Random Forest as a classifier and ANOVA as a feature selection method reached the best performance with an average of 82.1% accuracy, 75.9% specificity, 85.1% sensitivity, and 80.5% AUC. Overall, accuracy and AUC were the highest for predicting MCI to AD transition within 2 years (85% for accuracy as well as sensitivity and specificity).

### Selected Variables

Table 3 shows variables selected for transition models. Clinician’s judgment (the variable named COGSTAT in the NACC UDS V3 Data Dictionary) based on the neuropsychological examination was selected for all 4 transitions as one of the 20 variables. Supplemental Table 2 shows the distribution of responses for each variable by the transited vs. non-transited participants. Supplemental Table 2 can be read as follows: for example, the variable, COGSTAT (clinician’s judgement), was more likely to be coded as “1: better than normal for age” or “2: normal for age” at baseline for those who remained normal and the proportion of “3: one or two test scores abnormal” or “4: three or more scores are abnormal or lower than expected” increases as the duration of transition to conversion reduces from 3 years to 2 years, implying that those who converted to AD within a short duration showed more impairment at baseline, as expected. Transition to AD within 3 years was predicted mostly by neuropsychological tests and CDR (memory, community affairs and judgement subitems) while a transition to AD within 2 years was predicted more by variables indicating whether biomarkers including FDG-PET, amyloid PET, and CSF were assessed. We included a response variable that indicates these assessments were not conducted or unknown as a potential predictor variable, instead of removing it since the missing of this variable itself would be informative for predictions. 16% of those who progressed from MCI to AD within 2 years received FDG-PET assessment, while only 4% of those who did not transition to AD received the assessment (Supplemental Table 2.d). Interestingly, for the MCI2AD_2 transition, whether smoked a cigarette in the last 30 days was selected as one of the predictors, showing that 96% of those who remained MCI did not smoke while a lower % (72%) of those who transited to AD indicated they did not smoke.

### Probability threshold which optimizes prediction for pre-screening or study enrichment

**Figure 2.**
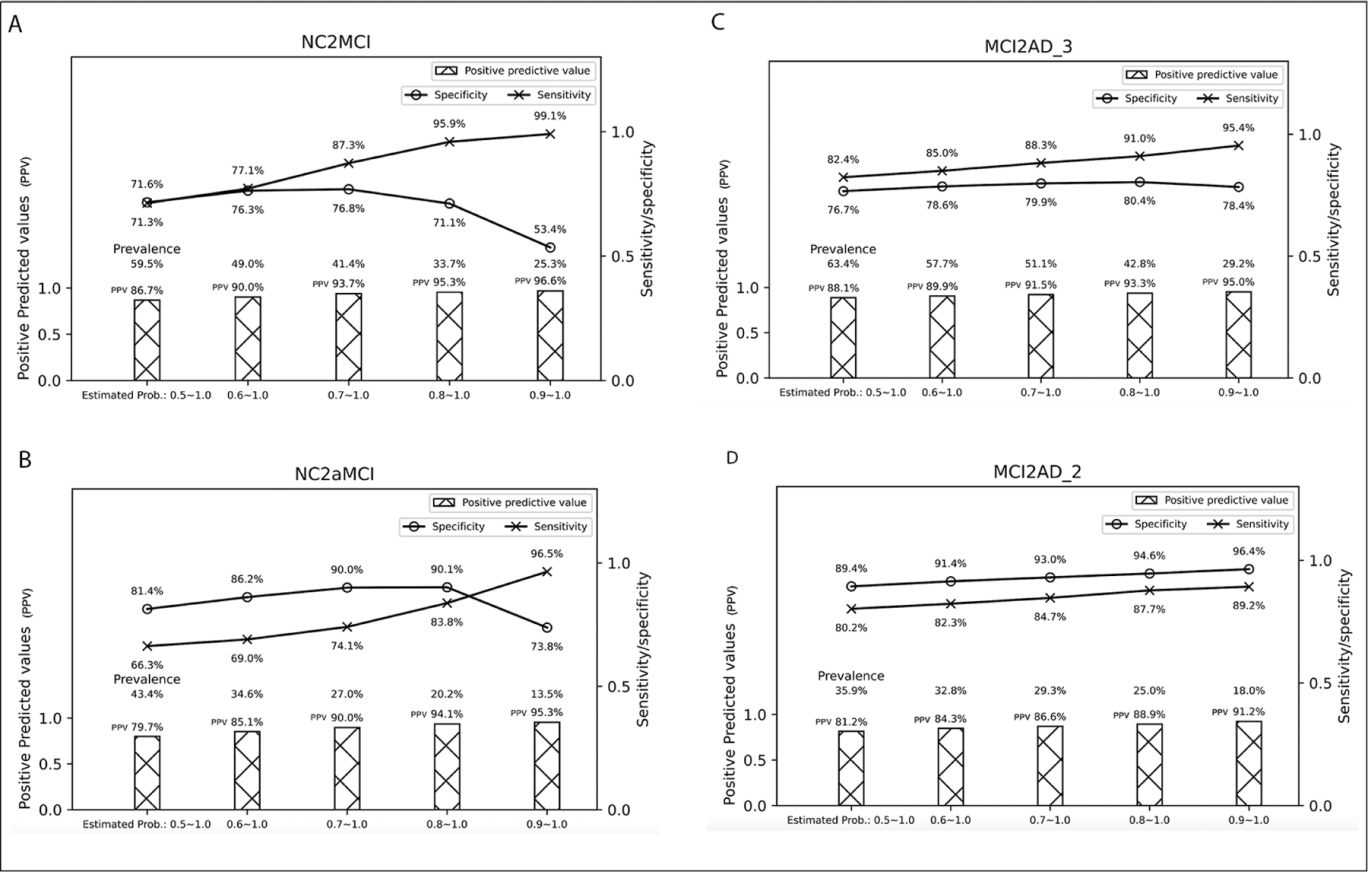
Estimated transitional probabilities and the associated sensitivity/specificity/positive predictive values with the proportion of subjects with sample estimated probabilities (based on the classifier Logistic Regression and number of features at 20) Note: For example in (A), if we select subjects with predicted probability ranging from 0.7 to 1.0, we can achieve a sensitivity of 87.3%, a positive predictive value of 93.7%, and 41.4 % of participants in the test datasets have this probability. On the other hand, if we select subjects with a predicted probability of 0.9 and above, we can achieve a sensitivity of 99.1%, a positive predictive value of 96.6%, but only 25.3% of the test data sets have this probability.

Although the higher the probability, the more likely the subject is going to transit, fewer subjects are selected as the probability increases. For example, based on our results, subjects with an estimated transitional probability of 0.9 (90%) for NC2MCI transition have a positive predictive value of 96.6%, indicating that they are almost guaranteed to transit to MCI within 4 years. However, a small fraction of our sample – 25.3% among the total samples used for NC2MCI prediction – has an estimated transitional probability of 0.9 or above. It is often a balance of how sure we would like to be in selecting those who progress in clinical symptoms vs. what proportion of subjects among the sampling pool can be selected for pre-screening or trial enrichment. We provided examples of sensitivity/specificity associated with each probability and how much % of subjects are selected (i.e., prevalence) in our total sampling pool in Figure. 2 (a–d). Examples of how to interpret the figures are described in the footnote.

### Calculator

**Figure 3.**
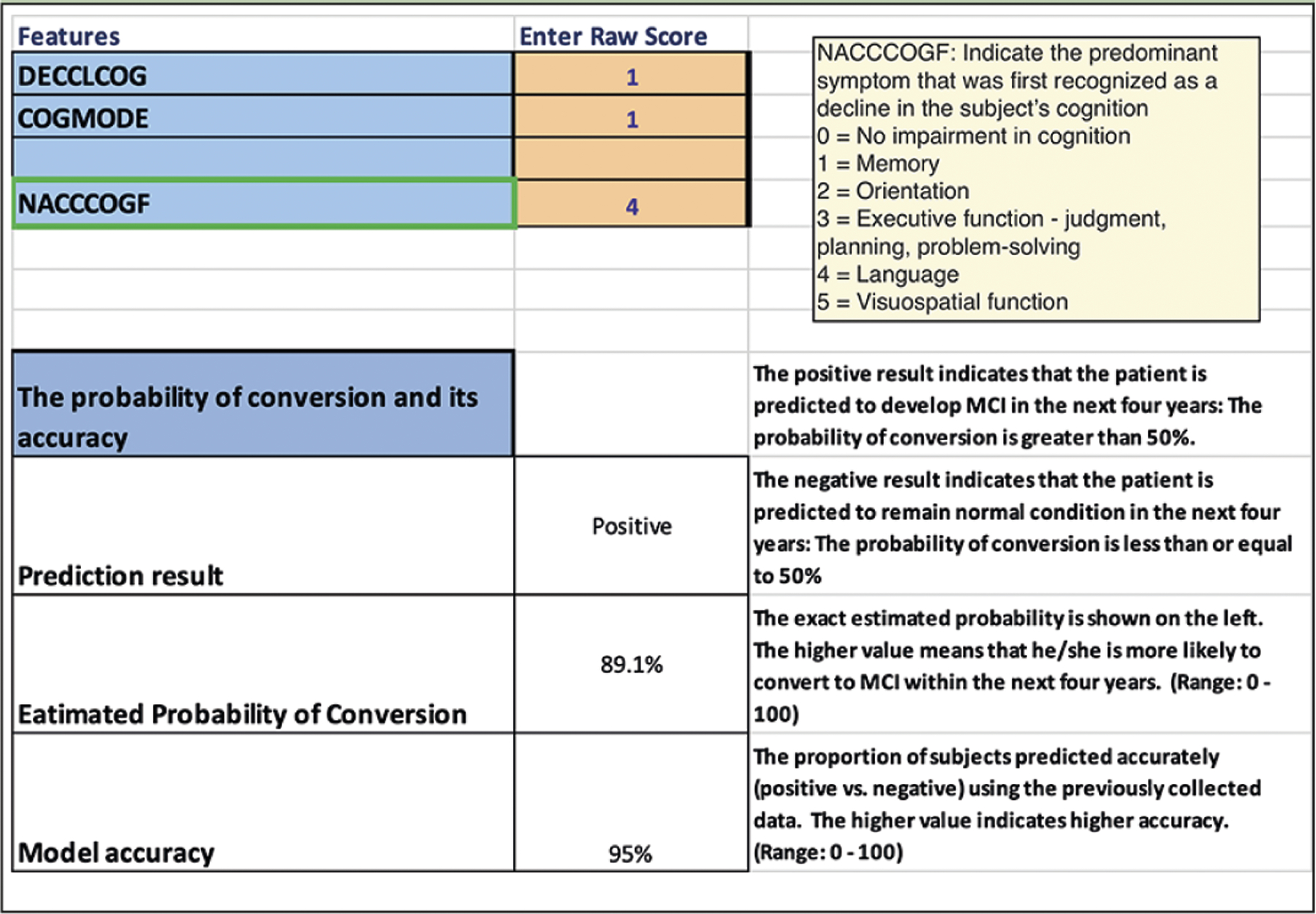
Example of how to use the calculator In the above example (screenshot), the excel sheet to estimate the probability of conversion from normal cognition to MCI within 4 years was selected. In this example, NACCCOGF variable (Clinician Judgment of Symptoms) was highlighted, and its response categories were shown in the pulldown menu. The variable names used in the calculator are the same as those used in the NACC code book so that the study coordinator can easily identify the targeted variable. The code book is located at: https://files.alz.washington.edu/documentation/uds3-rdd.pdf. In this example, given the data entered into this calculator, the subject is likely to convert to MCI within 4 years with 89.1% (the probability of the conversion). This probability is accurate with 95% of the simulated samples, implying that we have high confidence that this subject will convert to MCI within 4 years.

We developed a calculator that provides conversion probabilities by entering the person-specific information on the selected (in this study, 20) variables used in the NACC UDS V3. For each transition model, the hyper-parameters validated by the previous cross-validation were used to generate the prediction model. In order to generate probability output, we only used logistic regression as our classifier in the calculator. We applied bootstrap along with the classifier and dataset to estimate the mean value of the feature weights (i.e., the coefficient for each feature). And with the weights, we were able to estimate the probability of each new subject by using his or her information for the selected variables. The excel calculator can be downloaded at: https://www.shorturl.at/nHOY2. To aid in how to use the calculator, Figure 3 shows a screenshot of the calculator using the excel sheet used to estimate the probability of conversion from normal cognition to MCI within 4 years as an example. Please note that the pull-down menu functionality is available once it’s downloaded.

## Discussion

The main aim of this study is to generate prediction models (prediction in terms of clinical diagnosis) and a calculator to identify those at high risk of progressing from normal cognition to MCI, and from MCI to AD within relatively short follow-up durations. We used an observation window of 4 years from normal to MCI conversion and 2 and 3 years for MCI to AD conversions, i.e., a common duration of follow-up in pharmacological trials. Our user-friendly calculator can be used for pre-selecting subjects for further assessment of in-vivo biomarkers or for enriching study participants in various trials. The clinical variables selected are all derived from the data collected uniformly across all NIH-funded Alzheimer’s Disease Centers in the United States (UDS V3). In tables and supplemental materials, we included variable names used in the NACC Data Dictionary which is downloadable from the website (https://www.naccdata.org) so that researchers can replicate or expand our studies. Although the subjects used for this study are not from an epidemiological cohort and not are representative of the general elderly population in the US, these subjects are likely to represent those interested in participating in AD and related dementia (ADRD) clinical trials.

Similar to our previous study using an older UDS version (UDS V2) (5), neuropsychological test results played important roles in predictions: MoCA (global cognition), Craft story (learning and delayed memory), Animal Fluency (language, language-based executive functions), Trail Making A (psychomotor speed), Multilingual Naming Test (naming) and Benton Figure Delayed Drawing (visuospatial) are important predictors. Detailed descriptions of these tests are explained previously (29). As expected, participants’ reports of their subjective cognitive decline (DECSUB) played roles in predicting transitions to MCI, but informants’ reports were more important for predicting transitions to AD, consistent with prior studies of these transitions (21).

An intriguing finding is that the variables consistently predicting transitions involved clinicians’ judgements of participants’ degree of cognitive (either memory or overall) impairments. This variable was consistently selected as an important predictor, both in our previous study (2), and in other studies (22). Clinicians’ judgements synthesize information that might not be necessarily reported in the UDS, such as the way respondents answer questions, facial expressions and speech utterances, as well as detailed information collected from informants/partners. Although we do not believe that machine learning-based approaches can fully replace clinical judgements, it might be possible to develop algorithms that resemble clinical judgement using information not reported in standard paper-pencil formats. For example, a large number of studies using speech characteristics (linguistic and acoustic) showed promising results in differentiating early-stage MCI subjects from those with normal cognition (23–27). Additionally, information about longitudinal medical history free from recall bias might be supplemented by Electronic Health Records (EHR), using modeling approaches similar to those used in this and other studies (28).

Many approaches were proposed to predict conversions using only clinical variables, just like our current study (30–34). The analytical approaches and the model performance have been extensively discussed in each study. We would like to emphasize that the choice of a prediction model also depends on the nature of the data the developed model will be applied to in practice, and its usefulness depends on the available data. For example, Bernier et al. used longitudinal “slopes” of cognitive decline and its deviation from the normative decline to predict conversions (33, 34). Using only one global cognitive test is advantageous, but their chart requires at least two data points to obtain the slopes. Our calculator uses data obtained in NACC UDS V3, which takes about 90 minutes or longer to complete and requires trained assessors and clinicians, i.e., is tasking. Therefore, our approach is practical when patients come from ADCs or memory clinics where NACC UDS is routinely used. We anticipate that patients who are interested in enrolling in anti-amyloid and other drug trials are likely to be referred to memory clinics. The trial study coordinator can enter the data into the calculator to estimate the likelihood of clinical progression within a short interval for the study enrichment and/or pre-screening for biomarker assessments.

There are limitations to this study. Each AD Center enrolls participants in the NACC research cohort according to center-specific priorities. The cohort used in this study is not a representative group of the elderly population in the USA. The participants are skewed towards higher income and education strata. Certain racial and ethnic groups in the general population at risk of MCI or AD were also underrepresented. The generalizability of our results is limited and the probability calculator may not be applicable to other cohorts. We used linear models and thus captured only the linear relationships between the predictor variables and target variables in this study.

## Conclusion

Drawing on the most recent version (UDS V3) of uniformly collected data from all NIH-funded Alzheimer’s Disease Centers in the United States, we identified a small set of clinical variables which strongly predict transitions from normal cognition to MCI within 4 years and from MCI to AD within 2 and 3 years, updating our previous work (5). We developed a user-friendly conversion probability calculator that can be used for clinical trial enrichment and/or pre-screening for subsequent assessment of biological markers.

## Supplementary Material

Supplement

## Figures and Tables

**Table 1.** The number of sample size for each scenario: NC2MCI, NC2aMCI, MCI2AD_2, and MCI2AD_3

Transition	Total Sample Size: N of Unique Subjects	N with Transition (% of total)	N without Transition (% of total)*
NC2MCI Normal Cognition to MCI within 4 years	577	408 (70.7%)	169 (29.3%)
NC2aMCI Normal Cognition to aMCI within 4 years	352	183 (52.0%)	169 (48.0%)
MCI2AD_3 MCI to AD within 3 years	538	362 (67.3%)	176 (32.7%)
MCI2AD_2 MCI to AD within 2 years	882	321 (36.4%)	561 (63.6%)

*: Need to remain the normal cognitive status for either 4 years (NC2MCI, NC2aMCI), MCI for either 3 years (MCI2AD_3) or 2 years (MCI2AD_2).

**Table 2.** Overall Performance of Prediction Models for Each Transition

Transition*	Classifier	Feature selection	Accuracy	Specificity	Sensitivity	AUC
			mean±std			
NC2MCI	svm-rbf	InforGain	0.677 ± 0.054	0.659 ± 0.147	0.682 ± 0.087	0.670 ± 0.062
		Chi2	0.689 ± 0.054	0.692 ± 0.104	0.686 ± 0.073	0.689 ± 0.056
		Fisher	0.677 ± 0.050	0.618 ± 0.128	0.695 ± 0.101	0.657 ± 0.042
		ANOVA	0.693 ± 0.040	0.684 ± 0.120	0.696 ± 0.061	0.690 ± 0.053
		Lasso	0.663 ± 0.041	0.346 ± 0.104	0.787 ± 0.057	0.566 ± 0.050
		Tree-I	0.676 ± 0.044	0.521 ± 0.105	0.735 ± 0.068	0.628 ± 0.048
	RandomForest	InforGain	0.727 ± 0.052	0.452 ± 0.261	0.829 ± 0.143	0.641 ± 0.079
		Chi2	0.703 ± 0.055	0.480 ± 0.312	0.788 ± 0.169	0.634 ± 0.092
		Fisher	0.697 ± 0.052	0.273 ± 0.318	0.855 ± 0.194	0.564 ± 0.072
		ANOVA	0.763 ± 0.049	0.536 ± 0.163	0.848 ± 0.098	0.692 ± 0.059
		Lasso	0.722 ± 0.040	0.178 ± 0.188	0.930 ± 0.075	0.554 ± 0.067
		Tree-I	0.728 ± 0.037	0.254 ± 0.237	0.905 ± 0.116	0.580 ± 0.067
	**LR**	InforGain	0.697 ± 0.051	0.685 ± 0.126	0.700 ± 0.079	0.693 ± 0.059
		Chi2	0.684 ± 0.051	0.709 ± 0.147	0.674 ± 0.083	0.692 ± 0.064
		Fisher	0.661 ± 0.059	0.714 ± 0.106	0.638 ± 0.109	0.676 ± 0.045
		**ANOVA**	**0.715 ± 0.052**	**0.716 ± 0.117**	**0.713 ± 0.082**	**0.714 ± 0.054**
		Lasso	0.654 ± 0.062	0.519 ± 0.120	0.704 ± 0.089	0.611 ± 0.063
		Tree-I	0.662 ± 0.042	0.635 ± 0.119	0.671 ± 0.066	0.653 ± 0.052
NC2aMCI	svm-rbf	InforGain	0.690 ± 0.072	0.730 ± 0.112	0.653 ± 0.134	0.692 ± 0.071
		Chi2	0.671 ± 0.068	0.703 ± 0.132	0.648 ± 0.129	0.675 ± 0.069
		Fisher	0.623 ± 0.075	0.644 ± 0.154	0.603 ± 0.112	0.624 ± 0.077
		ANOVA	0.720 ± 0.057	0.783 ± 0.091	0.661 ± 0.143	0.722 ± 0.058
		Lasso	0.621 ± 0.079	0.681 ± 0.124	0.554 ± 0.135	0.617 ± 0.070
		Tree-I	0.661 ± 0.088	0.725 ± 0.129	0.598 ± 0.122	0.662 ± 0.086
	**RandomForest**	InforGain	0.711 ± 0.071	0.723 ± 0.193	0.687 ± 0.169	0.705 ± 0.066
		Chi2	0.662 ± 0.084	0.700 ± 0.254	0.607 ± 0.212	0.654 ± 0.079
		Fisher	0.633 ± 0.082	0.602 ± 0.293	0.641 ± 0.212	0.621 ± 0.079
		**ANOVA**	**0.746 ± 0.057**	**0.764 ± 0.120**	**0.716 ± 0.111**	**0.740 ± 0.055**
		Lasso	0.631 ± 0.074	0.534 ± 0.223	0.705 ± 0.199	0.620 ± 0.065
		Tree-I	0.681 ± 0.058	0.638 ± 0.215	0.700 ± 0.179	0.669 ± 0.061
	LR	InforGain	0.694 ± 0.077	0.762 ± 0.107	0.628 ± 0.114	0.695 ± 0.074
		Chi2	0.705 ± 0.060	0.749 ± 0.100	0.662 ± 0.120	0.706 ± 0.059
		Fisher	0.641 ± 0.075	0.703 ± 0.109	0.585 ± 0.113	0.644 ± 0.072
		ANOVA	0.735 ± 0.067	0.814 ± 0.080	0.663 ± 0.123	0.738 ± 0.064
		Lasso	0.633 ± 0.093	0.671 ± 0.114	0.588 ± 0.096	0.629 ± 0.088
		Tree-I	0.673 ± 0.064	0.737 ± 0.101	0.607 ± 0.118	0.672 ± 0.062
MCI2AD_3 (transition with 3 year)	svm-rbf	InforGain	0.796 ± 0.050	0.686 ± 0.162	0.848 ± 0.085	0.767 ± 0.065
		Chi2	0.795 ± 0.052	0.676 ± 0.150	0.852 ± 0.075	0.764 ± 0.063
		Fisher	0.746 ± 0.067	0.572 ± 0.163	0.828 ± 0.128	0.700 ± 0.059
		ANOVA	0.801 ± 0.060	0.718 ± 0.132	0.841 ± 0.084	0.779 ± 0.064
		Lasso	0.760 ± 0.044	0.542 ± 0.178	0.867 ± 0.078	0.704 ± 0.063
		Tree-I	0.743 ± 0.052	0.659 ± 0.166	0.784 ± 0.114	0.722 ± 0.055
	**RandomForest**	InforGain	0.817 ± 0.044	0.766 ± 0.159	0.842 ± 0.103	0.804 ± 0.054
		Chi2	0.817 ± 0.048	0.756 ± 0.165	0.846 ± 0.102	0.801 ± 0.060
		Fisher	0.737 ± 0.087	0.603 ± 0.241	0.805 ± 0.183	0.704 ± 0.078
		**ANOVA**	**0.821 ± 0.045**	**0.759 ± 0.144**	**0.851 ± 0.092**	**0.805 ± 0.051**
		Lasso	0.787 ± 0.058	0.583 ± 0.215	0.888 ± 0.092	0.735 ± 0.082
		Tree-I	0.789 ± 0.052	0.688 ± 0.164	0.840 ± 0.097	0.764 ± 0.060
	LR	InforGain	0.806 ± 0.043	0.767 ± 0.157	0.824 ± 0.077	0.795 ± 0.060
		Chi2	0.811 ± 0.041	0.750 ± 0.146	0.840 ± 0.062	0.795 ± 0.059
		Fisher	0.733 ± 0.063	0.681 ± 0.139	0.759 ± 0.114	0.720 ± 0.058
		ANOVA	0.806 ± 0.045	0.735 ± 0.140	0.840 ± 0.066	0.788 ± 0.058
		Lasso	0.777 ± 0.045	0.679 ± 0.148	0.826 ± 0.062	0.752 ± 0.064
		Tree-I	0.772 ± 0.059	0.746 ± 0.142	0.785 ± 0.096	0.765 ± 0.062
MCI2AD_2 (transition with 2 year)	svm-rbf	InforGain	0.853 ± 0.026	0.909 ± 0.044	0.759 ± 0.093	0.834 ± 0.032
		Chi2	0.849 ± 0.019	0.901 ± 0.047	0.761 ± 0.095	0.831 ± 0.028
		Fisher	0.731 ± 0.038	0.788 ± 0.079	0.629 ± 0.131	0.709 ± 0.047
		ANOVA	0.853 ± 0.021	0.886 ± 0.051	0.799 ± 0.097	0.843 ± 0.030
		Lasso	0.814 ± 0.021	0.901 ± 0.050	0.660 ± 0.110	0.781 ± 0.038
		Tree-I	0.815 ± 0.032	0.867 ± 0.051	0.731 ± 0.113	0.799 ± 0.043
	**RandomForest**	InforGain	0.841 ± 0.025	0.840 ± 0.048	0.847 ± 0.067	0.844 ± 0.027
		**Chi2**	**0.853 ± 0.028**	**0.856 ± 0.049**	**0.852 ± 0.066**	**0.854 ± 0.028**
		Fisher	0.705 ± 0.065	0.639 ± 0.160	0.811 ± 0.129	0.725 ± 0.035
		ANOVA	0.840 ± 0.030	0.845 ± 0.048	0.835 ± 0.073	0.840 ± 0.033
		Lasso	0.813 ± 0.037	0.813 ± 0.080	0.815 ± 0.085	0.814 ± 0.031
		Tree-I	0.811 ± 0.034	0.814 ± 0.082	0.803 ± 0.100	0.809 ± 0.031
	LR	InforGain	0.856 ± 0.021	0.899 ± 0.034	0.784 ± 0.074	0.841 ± 0.028
		Chi2	0.860 ± 0.023	0.894 ± 0.038	0.802 ± 0.081	0.848 ± 0.030
		Fisher	0.733 ± 0.044	0.730 ± 0.096	0.733 ± 0.100	0.732 ± 0.037
		ANOVA	0.857 ± 0.023	0.886 ± 0.040	0.807 ± 0.080	0.847 ± 0.029
		Lasso	0.834 ± 0.021	0.881 ± 0.034	0.749 ± 0.069	0.815 ± 0.029
		Tree-I	0.820 ± 0.028	0.853 ± 0.062	0.764 ± 0.089	0.809 ± 0.031

Note: mean and std (standard deviation) are calculated based on the repeated assessment process; The model which had the highest AUC was bolded.

**Table 3.** Selected Clinical Variables for Each Transition Model (Variables Names are Those Used in the National Alzheimer’s Coordinating Center (NACC) Data Dictionary

Features selected for: 1. NC2MCI, 2. NC2aMCI, 3. MCI2AD_3, 4. MCI2AD_4	Descriptions
COGSTAT : [1, 2, 3, 4]	Per clinician, based on the neuropsychological examination, the subject’s cognitive status is deemed
	0=Clinician unable to render opinion
	1=better than normal for age
	2=Normal for age
	3 = One or two test scores abnormal
	4=Three or more scores are abnormal or lower than expected
	9 = Missing
	−4=Not available: UDS form submitted did not collect data in this way, or a skip pattern precludes response to this question
COGMODE : [1, 2, 3]	Mode of onset of cognitive symptoms
	0=No impairment in cognition
	1 = Gradual
	2 = Subacute
	3 = Abrupt
	4 = Other (specify)
	99 = Unknown
HIPPATR : [1, 2, 4]	Hippocampal atrophy
	0 = No
	1 = Yes
	8 = Unknown/not assessed
	−4 = Not applicable: UDS form submitted did not collect data in this way, or a skip pattern precludes response to this question
TRAILA : [1, 2, 4] *	Trail Making Test Part A - Total number of seconds to complete
	0–150
	995=Physical problem
	996=Cognitive/behavior problem
	997=Other problem
	998=Verbal refusal
	−4=Not available: UDS form submitted did not collect data in this way, or a skip pattern precludes response to this question
DECIN : [1, 3, 4]	Does the co-participant report a decline in subject’s memory (relative to previously attained abilities)?
	0=No
	1=yes
	8=There is no co-participant
	9 = Unknown
JUDGMENT : [2, 3, 4]	Judgment and problem-solving
	0.0 = No impairment
	0.5 = Questionable impairment
	1.0 = Mild impairment
	2.0 = Moderate impairment
	3.0 = Severe impairment
NACCCOGF : [1, 2]	Indicate the predominant symptom that was first recognized as a decline in the subject’s cognition
	0=No impairment in cognition
	1 = Memory
	2 = Orientation
	3=Executive function - judgment, planning, problem-solving
	4 = Language
	5=Visuospatial function
	6 = Attention/concentration
	7=Fluctuating cognition
	8=Other (specify)
	99 = Unknown
NORMEXAM : [1, 2]	Were there abnormal neurological exam findings?
	0 = No abnormal findings
	1 = Yes - abnormal findings were consistent with syndromes listed in Questions 2–8
	2 = Yes - abnormal findings were consistent with age-associated changes or irrelevant to dementing disorders (e.g., bell’s palsy)
	−4 = Not available: UDS form submitted did not collect data in this way, or a skip pattern precludes response to this question
CRAFTVRS [1, 2, 3]	Craft Story 21 Recall (Immediate) - Total story units recalled, verbatim scoring
	0–44
	95=Physical problem
	96=Cognitive/behavior problem
	97=Other problem
	98=Verbal refusal
	−4 = Not available: UDS form submitted did not collect data in this way, or a skip pattern precludes response to this question
VEG : [1, 2]	Vegetables - Total number of vegetables named in 60 seconds
	0–77
	95=Physical problem
	96=Cognitive/behavior problem
	97=Other problem
	98=Verbal refusal
	−4 = Not available: UDS form submitted did not collect data in this way, or a skip pattern precludes response to this question
ANIMALS : [1, 2, 3]	Animals - Total number of animals named in 60 seconds
	0–77
	95=Physical problem
	96=Cognitive/behavior problem
	97=Other problem
	98=Verbal refusal
	−4 = Not available: UDS form submitted did not collect data in this way, or a skip pattern precludes response to this question
MINTTOTS [1, 2, 3]	Multilingual Naming Test (MINT) -Total score
	0–32
	95=Physical problem
	96=Cognitive/behavior problem
	97=Other problem
	98=Verbal refusal
	−4=Not available: UDS form submitted did not collect data in this way, or a skip pattern precludes response to this question
UDSBENTD : [2, 3, 4]	Total score for 10 to 15 minute delayed drawing of benson figure
	0–17
	95=Physical problem
	96=Cognitive/behavior problem
	97=Other problem
	98=Verbal refusal
	−4=Not available: UDS form submitted did not collect data in this way, or a skip pattern precludes response to this question
DECCLCOG : [1, 2]	based on the clinician’s judgment, is the subject currently experiencing meaningful impairment in cognition?
	0=No
	1 = Yes
	−4= Not available: UDS form submitted did not collect data in this way, or a skip pattern precludes response to this question
MOMODE : [1, 2]	Mode of onset of motor symptoms
	0=No motor symptoms
	1 = Gradual
	2 = Subacute
	3 = Abrupt
	4 = Other
	99 = Unknown
CRAFTDTI : [1, 2]	Craft Story 21 Recall (Delayed) - Delay time
	0–85
	99 = Unknown
	−4=Not available: UDS form submitted did not collect data in this way, or a skip pattern precludes response to this question
DECSUB : [1, 2]	Does the subject report a decline in memory (relative to previously attained abilities)?
	0=No
	1=Yes
	8=Could not be assessed/subject too impaired
	9= Unknown
	OTHCOG : [2, 3]	Presumptive etiologic diagnosis - Other neurological, genetic, or infectious
0 = No (assumed assessed and found not present)	
1 = Yes	
−4 = Not applicable: UDS form submitted did not collect data in this way, or a skip pattern precludes response to this question	
MEMORY : [3, 4]	Memory
	0.0 = No impairment
	0.5 = Questionable impairment
	1.0 = Mild impairment
	2.0 = Moderate impairment
COGORI : [3, 4]	Indicate whether the subject currently is meaningfully impaired, relative to previously attained abilities, in orientation
	0=No
	1 = Yes
	9 = Unknown
	−4= Not available: UDS form submitted did not collect data in this way, or a skip pattern precludes response to this question
COGJUDG : [3, 4]	Indicate whether the subject currently is meaningfully impaired, relative to previously attained abilities, in executive function - judgment, planning, or problem-solving
	0=No
	1 = Yes
	9 = Unknown
DEP : [3, 4]	Presumptive etiologic diagnosis - Depression
	0 = No (assumed assessed and found not present)
	1 = Yes
INDEPEND : [3, 4]	Level of independence
	1=Able to live independently
	2=Requires some assistance with complex activities
	3=Requires some assistance with basic activities
	4=Completely dependent
	9 = Unknown
COURSE : [3, 4]	Overall course of decline of cognitive/ behavioral/motor syndrome
	1=Gradually progressive
	2 = Stepwise
	3 = Static
	4 = Fluctuating
	5 = Improved
	8=Not applicable
	9 = Unknown
MEDSIF : [3, 4]	Primary, contributing, or non-contributing cause of cognitive impairment - medications
	1 = Primary
	2 = Contributing
	3 = Non-contributing
	7 = Cognitively impaired but no diagnosis of impairment due to medications
	8 = Diagnosis of normal cognition
	−4 = Not applicable: UDS form submitted did not collect data in this way, or a skip pattern precludes response to this question
COGVIS : [3, 4]	Indicate whether the subject currently is meaningfully impaired, relative to previously attained abilities, in visuospatial function
	0=No
	1 = Yes
	9 = Unknown
CDRLANG : [1]	Indicate whether the subject currently is meaningfully impaired, relative to previously attained abilities, in attention or concentration
	0=No
	1 = Yes
	9 = Unknown
NACCMOTF : [1]	Indicate the predominant symptom that was first recognized as a decline in the subject’s motor function
	0=No motor symptoms
	1=Gait disorder
	2 = Falls
	3=Tremor
	4 = Slowness
	99 = Unknown
BEMODE : [1]	Mode of onset of behavioral symptoms
	0=No behavioral symptoms
	1 = Gradual
	2 = Subacute
	3=Abrupt
	4=Other (specify)
	99 = Unknown
MOCATOTS : [1]	MoCA Total Raw Score - uncorrected
	0–30
	88=Item(s) or whole test not administered
	−4=Not available: UDS form submitted did not collect data in this way, or a skip pattern precludes response to this question
MOCAREGI : [1]	MoCA: Memory - Registration (two trials)
	0–10
	95=Physical problem 96=Cognitive/behavior problem 97=Other problem
	98=Verbal refusal
	−4=Not available: UDS form submitted did not collect data in this way, or a skip pattern precludes response to this question
DECCLMOT : [2]	based on the clinician’s judgment, is the subject currently experiencing any motor symptoms?
	0=No
	1 = Yes
	−4= Not available: UDS form submitted did not collect data in this way, or a skip pattern precludes response to this question
DIGBACCT : [2]	Number Span Test: backward - Number of correct trials
	0–14
	95=Physical problem 96=Cognitive/behavior problem 97=Other problem
	98=Verbal refusal
	−4=Not available: UDS form submitted did not collect data in this way, or a skip pattern precludes response to this question
NACCBEHF : [2]	Indicate the predominant symptom that was first recognized as a decline in the subject’s behavior
	0=No behavioral symptoms
	1 = Apathy/withdrawal
	2=Depressed mood
	3 = Psychosis
	4 = Disinhibition
	5 = Irritability
	6 = Agitation
	7=Personality change
	8=REM sleep behavior disorder
	9 = Anxiety
	10=Other (specify)
	99 = Unknown
COMMUN : [3]	Community affairs
	0.0 = No impairment
	0.5 = Questionable impairment
	1.0 = Mild impairment
	2.0 = Moderate impairment
	3.0 = Severe impairment
CORTIF : [3]	Primary, contributing, or non-contributing cause of cognitive impairment - Corticobasal degeneration (CbD)
	1 = Primary
	2 = Contributing
	3 = Non-contributing
	7 = Cognitively impaired but no CbD diagnosis
	8 = Diagnosis of normal cognition
COGMEM : [3]	Indicate whether the subject currently is meaningfully impaired, relative to previously attained abilities, in memory
	0=No
	1 = Yes
	9 = Unknown
TOBAC30 : [4]	Smoked cigarettes in last 30 days
	0=No
	1 = Yes
	9 = Unknown
	−4= Not available: UDS form submitted did not collect data in this way, or a skip pattern precludes response to this question
FDGAD : [4]	FDG-PET pattern of AD
	0 = No
	1 = Yes
	8 = Unknown/not assessed
	−4 = Not applicable: UDS form submitted did not collect data in this way, or a skip pattern precludes response to this question
AMYLPET : [4]	Abnormally elevated amyloid on PET
	0 = No
	1 = Yes
	8 = Unknown/not assessed
	−4 = Not applicable: UDS form submitted did not collect data in this way, or a skip pattern precludes response to this question
AMYLCSF : [4]	Abnormally low amyloid in CSF
	0 = No
	1 = Yes
	8 = Unknown/not assessed
	−4 = Not applicable: UDS form submitted did not collect data in this way, or a skip pattern precludes response to this question
NACCNE4S : [4]	Number of APOE e4 alleles
	0 = no e4 allele
	1 = 1 copy of e4 allele
	2 = 2 copies of e4 allele
	9 = missing/unknown/not assessed
HYPOSOM [4]	Hyposomnia/insomnia present
	0 = No
	1 = Yes
	8 = Not assessed
	−4 = Not available: UDS form submitted did not collect data in this way, or a skip pattern precludes response to this question
