# Supplementary material for: Predicting Progression from Normal to MCI and from MCI to AD Using Clinical Variables in the National Alzheimer’s Coordinating Center Uniform Data Set Version 3: Application of Machine Learning Models and a Probability Calculator
Source: J Prev Alzheimers Dis. Author manuscript; Available in PMC 2023 Mar 23. (PMC10033942; doi:10.14283/jpad.2023.10)
Supplement: Supplement [file NIHMS1876787-supplement-Supplement.docx]

**SUPPLEMENT A**

Coding Algorithm for Clinical Diagnoses and Etiology.

The following variables were used to define the cognitive status and etiology (normal, MCI, aMCI and dementia due to AD): CDRGLOB, NACCTMCI, NACCUDSD and NACCALZP.

In the MCI conversion prediction task, the incidence of MCI was defined as CDRGLOB >= 0.5, which includes those who transitioned directly from normal to AD (i.e., transited directly to AD without MCI diagnosis), assuming that they did experience MCI before their transition to AD. Incidence of aMCI was identified when CDRGLOB >= 0.5 and NACCTMCI = 1 or 2. The incidence of naMCI was defined as CDRGLOB >= 0.5 and NACCTMCI = 3 or 4.

Incidence of AD was defined as CDRGLOB = 1 or 2 or 3 and NACCUDSD = 4 and NACCALZP = 1. **Supplement Table 1** below explains these variables.

**Supplement Table 1:** UDS V3 variables used to determine diagnoses and etiology (AD).

| Labeling variables | Short descriptor | Allowable codes |
| --- | --- | --- |
| CDRGLOB | Global CDR | 0.0 = No impairment  0.5 = Questionable impairment  1.0 = Mild impairment  2.0 = Moderate impairment  3.0 = Severe impairment |
| NACCTMCI | Mild cognitive impairment (MCI) type | 1 = Amnestic MCI- single domain  2 = Amnestic MCI- multiple domain  3 = Non-amnestic MCI- single domain  4 = Non-amnestic MCI- multiple domain  8 = No diagnosis of MCI |
| NACCUDSD | Cognitive status at UDS visit | 1 = Normal cognition  2 = Impaired-not-MCI  3 = MCI  4 = Dementia |
| NACCALZP | Primary, contributing, or non-contributing cause of observed cognitive impairment - Alzheimer’s disease (AD) | 1 = Primary  2 = Contributing  3 = Non-contributing  7 = Cognitively impaired but not AD diagnosis  8 = Diagnosis of normal cognition |

**Supplement Table 2**. Descriptive statistics of predictor variables

a: NC2MCI.

| **Features Selected for NC2MCI** | **In total sample** | | | **In the sample who developed MCI** | **In the sample who retained NC** | **Descriptions** |
| --- | --- | --- | --- | --- | --- | --- |
|  | **Proportion in each response** | | | | |  |
| DECCLCOG | 0: 0.79  1: 0.21 | | | 0: 0.71  1: 0.29 | 0: 0.99  1: 0.01 | based on the clinician’s judgment, is the subject currently experiencing meaningful impairment in cognition?  0=No  1 = Yes  -4= Not available: UDS form submitted did not collect data in this way, or a skip pattern precludes response to this question |
| COGMODE | 0: 0.79  1: 0.18  2: 0.01  3: 0.00  4: 0.00  99: 0.02 | | | 0: 0.71  1: 0.24  2: 0.01  3: 0.00  4: 0.00  99: 0.03 | 0: 0.99  1: 0.01  2: 0.00  3: 0.00  4: 0.00  99: 0.00 | Mode of onset of cognitive symptoms  0=No impairment in cognition  1 = Gradual  2 = Subacute  3 = Abrupt  4 = Other (specify)  99 = Unknown |
| NACCCOGF | 0: 0.79  1: 0.08  2: 0.01  3: 0.03  4: 0.07  5: 0.01  6: 0.01  8: 0.00  99: 0.01 | | | 0: 0.71  1: 0.11  2: 0.00  3: 0.04  4: 0.10  5: 0.01  6: 0.02  8: 0.00  99: 0.01 | 0: 0.99  1: 0.00  2: 0.01  3: 0.01  4: 0.00  5: 0.00  6: 0.00  8: 0.00  99: 0.00 | Indicate the predominant symptom that was first recognized as a decline in the subject’s cognition  0=No impairment in cognition  1 = Memory  2 = Orientation  3=Executive function - judgment, planning, problem-solving  4 = Language  5=Visuospatial function  6 = Attention/concentration  7=Fluctuating cognition  8=Other (specify)  99 = Unknown |
| NORMEXAM | 0: 0.77  1: 0.06  2: 0.17 | | | 0: 0.70  1: 0.08  2: 0.22 | 0: 0.93  1: 0.02  2: 0.05 | Were there abnormal neurological exam findings?  0 = No abnormal findings  1 = Yes - abnormal findings were consistent with syndromes listed in Questions 2-8  2 = Yes - abnormal findings were consistent with age-associated changes or irrelevant to dementing disorders (e.g., bell’s palsy)  -4 = Not available: UDS form submitted did not collect data in this way, or a skip pattern precludes response to this question |
| COGSTAT | 0: 0.20  1: 0.05  2: 0.37  3: 0.25  4: 0.13 | | | 0: 0.19  1: 0.03  2: 0.31  3: 0.29  4: 0.18 | 0: 0.21  1: 0.10  2: 0.52  3: 0.15  4: 0.01 | Per clinician, based on the neuropsychological examination, the subject’s cognitive status is deemed  0=Clinician unable to render opinion  1=better than normal for age  2=Normal for age  3 = One or two test scores abnormal  4=Three or more scores are abnormal or lower than expected  9 = Missing  -4=Not available: UDS form submitted  did not collect data in this way, or a skip pattern precludes response to this question |
| HIPPATR | 0: 0.25  1: 0.06  8: 0.69 | | | 0: 0.30  1: 0.08  8: 0.62 | 0: 0.13  1: 0.01  8: 0.86 | Hippocampal atrophy  0 = No  1 = Yes  8 = Unknown/not assessed  -4 = Not applicable: UDS form submitted  did not collect data in this way, or a skip pattern precludes response to this question |
| MOMODE | 0: 0.93  1: 0.07  4: 0.00  99: 0.00 | | | 0: 0.90  1: 0.09  4: 0.00  99: 0.00 | 0: 1.00  1: 0.00  4: 0.00  99: 0.00 | Mode of onset of motor symptoms  0=No motor symptoms  1 = Gradual  2 = Subacute  3 = Abrupt  4 = Other  99 = Unknown |
| CDRLANG | 0: 0.91  0: 0.06  1: 0.02  2: 0.01  3: 0.00 | | | 0: 0.88  0: 0.07  1: 0.03  2: 0.01  3: 0.00 | 0: 0.99  0: 0.01  1: 0.00  2: 0.00  3: 0.00 | Indicate whether the subject currently  is meaningfully impaired, relative to previously attained abilities, in attention or concentration  0=No  1=Yes  9 = Unknown |
| NACCMOTF | 0: 0.93  1: 0.02  2: 0.01  3: 0.03  4: 0.01  99: 0.00 | | | 0: 0.90  1: 0.02  2: 0.01  3: 0.04  4: 0.02  99: 0.00 | 0: 1.00  1: 0.00  2: 0.00  3: 0.00  4: 0.00  99: 0.00 | Indicate the predominant symptom that was first recognized as a decline in the subject’s motor function  0=No motor symptoms  1=Gait disorder  2 = Falls  3=Tremor  4 = Slowness  99 = Unknown |
| DECIN | 0: 0.78  1: 0.17  8: 0.05 | | | 0: 0.75  1: 0.21  8: 0.04 | 0: 0.86  1: 0.08  8: 0.07 | Does the co-participant report a decline in subject’s memory (relative to previously attained abilities)?  0=No  1=Yes  8=There is no co-participant  9 = Unknown |
| DECSUB | 0: 0.70  1: 0.30  8: 0.00 | | | 0: 0.65  1: 0.35  8: 0.00 | 0: 0.80  1: 0.20  8: 0.00 | Does the subject report a decline in memory (relative to previously attained abilities)?  0=No  1=Yes  8=Could not be assessed/subject too  impaired  9= Unknown |
| BEMODE | 0: 0.82  1: 0.14  2: 0.01  3: 0.01  4: 0.01  99: 0.01 | | | 0: 0.79  1: 0.17  2: 0.01  3: 0.01  4: 0.01  99: 0.01 | 0: 0.91  1: 0.06  2: 0.00  3: 0.00  4: 0.02  99: 0.01 | Mode of onset of behavioral symptoms  0=No behavioral symptoms  1 = Gradual  2 = Subacute  3=Abrupt  4=Other (specify)  99 = Unknown |
| **Continuous Variables** | **In total sample** | | | **In the sample who developed MCI** | **In the sample who retained NC** | **Descriptions** |
|  | **Min. value** | **Max. value** | **Mean** | **Mean** | **Mean** |  |
| MOCATOTS | 9.0 | 30.0 | 24.9 | 24.2 | 26.3 | MoCA Total Raw Score - uncorrected  0–30  88=Item(s) or whole test not administered  -4=Not available: UDS form submitted did not collect data in this way, or a skip pattern precludes response to this question |
| CRAFTVRS | 0.0 | 36.0 | 19.2 | 17.8 | 22.2 | Craft Story 21 Recall (Immediate) - Total story units recalled, verbatim scoring  0-44  95=Physical problem  96=Cognitive/behavior problem  97=Other problem  98=Verbal refusal  -4 = Not available: UDS form submitted did not collect data in this way, or a skip pattern precludes response to this question |
| VEG | 1.0 | 26.0 | 13.5 | 12.8 | 15.2 | Vegetables - Total number of vegetables named in 60 seconds  0-77  95=Physical problem 96=Cognitive/behavior problem 97=Other problem  98=Verbal refusal  -4 = Not available: UDS form submitted did not collect data in this way, or a skip pattern precludes response to this question |
| ANIMALS | 4.0 | 49.0 | 19.4 | 18.3 | 21.9 | Animals - Total number of animals named in 60 seconds  0-77  95=Physical problem  96=Cognitive/behavior problem  97=Other problem  98=Verbal refusal  -4 = Not available: UDS form submitted did not collect data in this way, or a skip pattern precludes response to this question |
| TRAILA | 13.0 | 150.0 | 35.9 | 38.1 | 30.7 | Trail Making Test Part A - Total number of seconds to complete  0–150  995=Physical problem  996=Cognitive/behavior problem  997=Other problem  998=Verbal refusal  -4=Not available: UDS form submitted did not collect data in this way, or a skip pattern precludes response to this question |
| CRAFTDTI | 8.0 | 35.0 | 18.1 | 18.5 | 17.3 | Craft Story 21 Recall (Delayed) - Delay time  0-85  99 = Unknown  -4=Not available: UDS form submitted did not collect data in this way, or a skip pattern precludes response to this question |
| MOCAREGI | 0.0 | 10.0 | 9.1 | 9.0 | 9.5 | MoCA: Memory - Registration (two trials)  0–10  95=Physical problem 96=Cognitive/behavior problem 97=Other problem  98=Verbal refusal  -4=Not available: UDS form submitted did not collect data in this way, or a skip pattern precludes response to this question |
| MINTTOTS | 1.0 | 32.0 | 29.0 | 28.6 | 30.0 | Multilingual Naming Test (MINT) -Total score  0-32  95=Physical problem  96=Cognitive/behavior problem  97=Other problem  98=Verbal refusal  -4=Not available: UDS form submitted  did not collect data in this way, or a skip pattern precludes response to this question |

**b:** NC2aMCI.

| **Features Selected for NC2aMCI** | **In total sample** | | | **In the sample who developed aMCI** | **In the sample who retained NC** | **Descriptions** |
| --- | --- | --- | --- | --- | --- | --- |
|  | **Proportion in each response** | | | | |  |
| DECCLCOG | 0: 0.84  1: 0.16 | | | 0: 0.71  1: 0.29 | 0: 0.99  1: 0.01 | based on the clinician’s judgment, is the subject currently experiencing meaningful impairment in cognition?  0=No  1 = Yes  -4= Not available: UDS form submitted did not collect data in this way, or a skip pattern precludes response to this question |
| COGMODE | 0: 0.84  1: 0.13  2: 0.01  4: 0.00  99: 0.02 | | | 0: 0.71  1: 0.23  2: 0.01  4: 0.01  99: 0.04 | 0: 0.99  1: 0.01  2: 0.00  4: 0.00  99: 0.00 | Mode of onset of cognitive symptoms  0=No impairment in cognition  1 = Gradual  2 = Subacute  3 = Abrupt  4 = Other (specify)  99 = Unknown |
| NACCCOGF | 0: 0.84  1: 0.08  2: 0.01  3: 0.03  4: 0.02  5: 0.01  6: 0.01  99: 0.00 | | | 0: 0.71  1: 0.15  2: 0.01  3: 0.05  4: 0.04  5: 0.02  6: 0.01  99: 0.01 | 0: 0.99  1: 0.00  2: 0.01  3: 0.01  4: 0.00  5: 0.00  6: 0.00  99: 0.00 | Indicate the predominant symptom that was first recognized as a decline in the subject’s cognition  0=No impairment in cognition  1 = Memory  2 = Orientation  3=Executive function - judgment, planning, problem-solving  4 = Language  5=Visuospatial function  6 = Attention/concentration  7=Fluctuating cognition  8=Other (specify)  99 = Unknown |
| COGSTAT | 0: 0.19  1: 0.05  2: 0.39  3: 0.24  4: 0.13 | | | 0: 0.17  1: 0.01  2: 0.28  3: 0.31  4: 0.23 | 0: 0.21  1: 0.10  2: 0.52  3: 0.15  4: 0.01 | Per clinician, based on the neuropsychological examination, the subject’s cognitive status is deemed  0=Clinician unable to render opinion 1=better than normal for age 2=Normal for age  3 = One or two test scores abnormal 4=Three or more scores are abnormal or lower than expected  9 = Missing  -4=Not available: UDS form submitted did not collect data in this way, or a skip pattern precludes response to this question |
| NORMEXAM | 0: 0.83  1: 0.03  2: 0.14 | | | 0: 0.73  1: 0.04  2: 0.22 | 0: 0.93  1: 0.02  2: 0.05 | Were there abnormal neurological exam findings?  0 = No abnormal findings  1 = Yes - abnormal findings were consistent with syndromes listed in Questions 2-8  2 = Yes - abnormal findings were consistent with age-associated changes or irrelevant to dementing disorders (e.g., bell’s palsy)  -4 = Not available: UDS form submitted did not collect data in this way, or a skip pattern precludes response to this question |
| HIPPATR | 0: 0.19  1: 0.05  8: 0.76 | | | 0: 0.25  1: 0.08  8: 0.67 | 0: 0.13  1: 0.01  8: 0.86 | Hippocampal atrophy  0 = No  1 = Yes  8 = Unknown/not assessed  -4 = Not applicable: UDS form submitted did not collect data in this way, or a skip pattern precludes response to this question |
| DECCLMOT | 0: 0.96  1: 0.04 | | | 0: 0.92  1: 0.08 | 0: 1.00  1: 0.00 | based on the clinician’s judgment, is the subject currently experiencing any motor symptoms?  0=No  1 = Yes  -4= Not available: UDS form submitted did not collect data in this way, or a skip pattern precludes response to this question |
| MOMODE | 0: 0.96  1: 0.03  4: 0.00  99: 0.00 | | | 0: 0.92  1: 0.07  4: 0.01  99: 0.01 | 0: 1.00  1: 0.00  4: 0.00  99: 0.00 | Mode of onset of motor symptoms  0=No motor symptoms  1 = Gradual  2 = Subacute  3 = Abrupt  4 = Other  99 = Unknown |
| DECSUB | 0: 0.72  1: 0.28 | | | 0: 0.64  1: 0.36 | 0: 0.80  1: 0.20 | Does the subject report a decline in memory (relative to previously attained abilities)?  0=No  1=Yes  8=Could not be assessed/subject too  impaired  9= Unknown |
| JUDGMENT | 0.0: 0.94  0.5: 0.06 | | | 0.0: 0.90  0.5: 0.10 | 0.0: 0.98  0.5: 0.02 | Judgment and problem-solving  0.0 = No impairment  0.5 = Questionable impairment  1.0 = Mild impairment  2.0 = Moderate impairment  3.0 = Severe impairment |
| OTHCOG | 0: 0.94  1: 0.06 | | | 0: 0.91  1: 0.09 | 0: 0.98  1: 0.02 | Presumptive etiologic diagnosis - Other neurological, genetic, or infectious  0 = No (assumed assessed and found  not present)  1 = Yes  -4 = Not applicable: UDS form submitted did not collect data in this way, or a skip pattern precludes response to this question |
| NACCBEHF | 0: 0.86  1: 0.01  2: 0.06  5: 0.02  6: 0.00  8: 0.01  9: 0.03  10: 0.01  99: 0.00 | | | 0: 0.81  1: 0.01  2: 0.07  5: 0.04  6: 0.00  8: 0.01  9: 0.05  10: 0.01  99: 0.01 | 0: 0.91  1: 0.01  2: 0.06  5: 0.00  6: 0.01  8: 0.01  9: 0.01  10: 0.00  99: 0.00 | Indicate the predominant symptom that was first recognized as a decline in the subject’s behavior  0=No behavioral symptoms  1 = Apathy/withdrawal  2=Depressed mood  3 = Psychosis  4 = Disinhibition  5 = Irritability  6 = Agitation  7=Personality change  8=REM sleep behavior disorder  9 = Anxiety  10=Other (specify)  99 = Unknown |
| **Continuous variables** | **In total sample** | | | **In the sample who developed aMCI** | **In the sample who retained NC** | **Descriptions** |
|  | **Min. value** | **Max. value** | **Mean** | **Mean** | **Mean** |  |
| CRAFTVRS | 0.0 | 36.0 | 19.3 | 16.5 | 22.2 | Craft Story 21 Recall (Immediate) - Total story units recalled, verbatim scoring  0-44  95=Physical problem 96=Cognitive/behavior problem 97=Other problem  98=Verbal refusal  -4 = Not available: UDS form submitted did not collect data in this way, or a skip pattern precludes response to this question |
| ANIMALS | 5.0 | 49.0 | 19.6 | 17.5 | 21.9 | Animals - Total number of animals named in 60 seconds  0-77  95=Physical problem 96=Cognitive/behavior problem 97=Other problem  98=Verbal refusal  -4 = Not available: UDS form submitted did not collect data in this way, or a skip pattern precludes response to this question |
| VEG | 1.0 | 26.0 | 13.7 | 12.4 | 15.2 | Vegetables - Total number of vegetables named in 60 seconds  0-77  95=Physical problem 96=Cognitive/behavior problem 97=Other problem  98=Verbal refusal  -4 = Not available: UDS form submitted did not collect data in this way, or a skip pattern precludes response to this question |
| CRAFTDTI | 8.0 | 30.0 | 18.0 | 18.7 | 17.3 | Craft Story 21 Recall (Delayed) - Delay time  0-85  99 = Unknown  -4=Not available: UDS form submitted did not collect data in this way, or a skip pattern precludes response to this question |
| UDSBENTD | 1.0 | 17.0 | 10.3 | 9.6 | 11.1 | Total score for 10 to 15 minute delayed drawing of benson figure  0–17  95=Physical problem  96=Cognitive/behavior problem  97=Other problem  98=Verbal refusal  -4=Not available: UDS form submitted did not collect data in this way, or a skip pattern precludes response to this question |
| TRAILA | 14.0 | 144.0 | 35.1 | 39.3 | 30.7 | Trail Making Test Part A - Total number of seconds to complete  0–150  995=Physical problem 996=Cognitive/behavior problem 997=Other problem  998=Verbal refusal  -4=Not available: UDS form submitted did not collect data in this way, or a skip pattern precludes response to this question |
| DIGBACCT | 0.0 | 13.0 | 6.3 | 5.9 | 6.8 | Number Span Test: backward - Number of correct trials  0–14  95=Physical problem 96=Cognitive/behavior problem 97=Other problem  98=Verbal refusal  -4=Not available: UDS form submitted did not collect data in this way, or a skip pattern precludes response to this question |
| MINTTOTS | 4.0 | 32.0 | 29.2 | 28.4 | 30.0 | Multilingual Naming Test (MINT) -Total score  0-32  95=Physical problem  96=Cognitive/behavior problem  97=Other problem  98=Verbal refusal  -4=Not available: UDS form submitted  did not collect data in this way, or a skip pattern precludes response to this question |

c: MCI2AD_3.

| **Features Selected for MCI2AD_3** | **In total sample** | | | **In the sample who developed AD** | **In sample who retained MCI** | **Descriptions** |
| --- | --- | --- | --- | --- | --- | --- |
|  | **Proportion in each response** | | | | |  |
| MEMORY | 0.0: 0.04  0.5: 0.46  1.0: 0.50  2.0: 0.01  3.0: 0.00 | | | 0.0: 0.02  0.5: 0.30  1.0: 0.67  2.0: 0.01  3.0: 0.00 | 0.0: 0.09  0.5: 0.77  1.0: 0.14  2.0: 0.00  3.0: 0.00 | Memory  0.0 = No impairment  0.5 = Questionable impairment  1.0 = Mild impairment  2.0 = Moderate impairment  3.0 = Severe impairment |
| COMMUN | 0.0: 0.41  0.5: 0.51  1.0: 0.09 | | | 0.0: 0.25  0.5: 0.63  1.0: 0.12 | 0.0: 0.74  0.5: 0.25  1.0: 0.01 | Community affairs  0.0 = No impairment  0.5 = Questionable impairment  1.0 = Mild impairment  2.0 = Moderate impairment  3.0 = Severe impairment |
| JUDGMENT | 0.0: 0.23  0.5: 0.54  1.0: 0.22 | | | 0.0: 0.12  0.5: 0.58  1.0: 0.30 | 0.0: 0.47  0.5: 0.47  1.0: 0.06 | Judgment and problem-solving  0.0 = No impairment  0.5 = Questionable impairment  1.0 = Mild impairment  2.0 = Moderate impairment  3.0 = Severe impairment |
| COGJUDG | 0: 0.34  1: 0.66  9: 0.00 | | | 0: 0.20  1: 0.80  9: 0.00 | 0: 0.62  1: 0.38  9: 0.01 | Indicate whether the subject currently is meaningfully impaired, relative to previously attained abilities, in executive function - judgment, planning, or problem-solving  0=No  1=Yes  9 = Unknown |
| COGORI | 0: 0.53  1: 0.47  9: 0.00 | | | 0: 0.38  1: 0.62  9: 0.00 | 0: 0.84  1: 0.16  9: 0.01 | Indicate whether the subject currently is meaningfully impaired, relative to previously attained abilities, in orientation  0=No  1 = Yes  9 = Unknown  -4= Not available: UDS form submitted did not collect data in this way, or a skip pattern precludes response to this question |
| DEP | 0: 0.81  1: 0.19 | | | 0: 0.82  1: 0.18 | 0: 0.81  1: 0.19 | Presumptive etiologic diagnosis - Depression  0 = No (assumed assessed and found not present)  1 = Yes |
| INDEPEND | 1: 0.53  2: 0.41  3: 0.04  4: 0.00  9: 0.02 | | | 1: 0.39  2: 0.53  3: 0.06  4: 0.00  9: 0.02 | 1: 0.82  2: 0.18  3: 0.00  4: 0.00  9: 0.00 | Level of independence  1=Able to live independently  2=Requires some assistance with complex activities  3=Requires some assistance with basic  activities  4=Completely dependent  9 = Unknown |
| COGSTAT | 0: 0.17  1: 0.00  2: 0.04  3: 0.22  4: 0.57 | | | 0: 0.16  1: 0.00  2: 0.01  3: 0.14  4: 0.69 | 0: 0.19  1: 0.01  2: 0.10  3: 0.38  4: 0.33 | Per clinician, based on the neuropsychological examination, the subject’s cognitive status is deemed  0=Clinician unable to render opinion 1=better than normal for age 2=Normal for age  3 = One or two test scores abnormal 4=Three or more scores are abnormal or lower than expected  9 = Missing  -4=Not available: UDS form submitted did not collect data in this way, or a skip pattern precludes response to this question |
| MEDSIF | 1: 0.00  2: 0.00  7: 0.96  8: 0.03 | | | 1: 0.00  2: 0.00  7: 0.99  8: 0.00 | 1: 0.01  2: 0.01  7: 0.89  8: 0.10 | Primary, contributing, or non-contributing cause of cognitive impairment - medications  1 = Primary  2 = Contributing  3 = Non-contributing  7 = Cognitively impaired but no diagnosis of impairment due to medications  8 = Diagnosis of normal cognition  -4 = Not applicable: UDS form submitted did not collect data in this way, or a skip pattern precludes response to this question |
| CORTIF | 1: 0.00  2: 0.00  7: 0.96  8: 0.03 | | | 1: 0.00  2: 0.00  7: 0.99  8: 0.00 | 1: 0.01  2: 0.00  7: 0.90  8: 0.10 | Primary, contributing, or non-contributing cause of cognitive impairment - Corticobasal degeneration (CbD)  1 = Primary  2 = Contributing  3 = Non-contributing  7 = Cognitively impaired but no CbD  diagnosis  8 = Diagnosis of normal cognition |
| COURSE | 1: 0.91  2: 0.01  3: 0.04  4: 0.00  5: 0.00  8: 0.02  9: 0.02 | | | 1: 0.97  2: 0.01  3: 0.01  4: 0.00  5: 0.00  8: 0.00  9: 0.01 | 1: 0.78  2: 0.01  3: 0.09  4: 0.01  5: 0.01  8: 0.05  9: 0.06 | Overall course of decline of cognitive/ behavioral/motor syndrome  1=Gradually progressive  2 = Stepwise  3 = Static  4 = Fluctuating  5 = Improved  8=Not applicable  9 = Unknown |
| COGMEM | 0: 0.06  1: 0.94 | | | 0: 0.02  1: 0.98 | 0: 0.14  1: 0.86 | Indicate whether the subject currently is meaningfully impaired, relative to previously attained abilities, in memory  0=No  1=Yes  9 = Unknown |
| DECIN | 0: 0.06  1: 0.93  8: 0.01 | | | 0: 0.02  1: 0.97  8: 0.01 | 0: 0.12  1: 0.85  8: 0.02 | Does the co-participant report a decline in subject’s memory (relative to previously attained abilities)?  0=No  1=Yes  8=There is no co-participant  9 = Unknown |
| COGVIS | 0: 0.74  1: 0.25  9: 0.01 | | | 0: 0.68  1: 0.31  9: 0.01 | 0: 0.88  1: 0.11  9: 0.01 | Indicate whether the subject currently is meaningfully impaired, relative to previously attained abilities, in visuospatial function  0=No  1=Yes  9 = Unknown |
| OTHCOG | 0: 0.96  1: 0.04 | | | 0: 0.99  1: 0.01 | 0: 0.90  1: 0.10 | Presumptive etiologic diagnosis - Other neurological, genetic, or infectious  0 = No (assumed assessed and found  not present)  1 = Yes  -4 = Not applicable: UDS form submitted did not collect data in this way, or a skip pattern precludes response to this question |
| COGMODE | 0: 0.01  1: 0.95  2: 0.01  3: 0.01  4: 0.00  99: 0.02 | | | 0: 0.00  1: 0.98  2: 0.01  3: 0.01  4: 0.00  99: 0.01 | 0: 0.03  1: 0.89  2: 0.01  3: 0.03  4: 0.01  99: 0.03 | Mode of onset of cognitive symptoms  0=No impairment in cognition  1 = Gradual  2 = Subacute  3 = Abrupt  4 = Other (specify)  99 = Unknown |
| **Continuous Variables** | **In total sample** | | | **In the sample who developed AD** | **In the sample who retained MCI** | **Descriptions** |
|  | **Min. value** | **Max. value** | **Mean** | **Mean** | **Mean** |  |
| CRAFTVRS | 0.0 | 35.0 | 12.1 | 9.7 | 17.0 | Craft Story 21 Recall (Immediate) - Total story units recalled, verbatim scoring  0-44  95=Physical problem  96=Cognitive/behavior problem  97=Other problem  98=Verbal refusal  -4 = Not available: UDS form submitted did not collect data in this way, or a skip pattern precludes response to this question |
| MINTTOTS | 1.0 | 32.0 | 27.0 | 26.0 | 29.0 | Multilingual Naming Test (MINT) -Total score  0-32  95=Physical problem  96=Cognitive/behavior problem  97=Other problem  98=Verbal refusal  -4=Not available: UDS form submitted  did not collect data in this way, or a skip pattern precludes response to this question |
| ANIMALS | 0.0 | 36.0 | 14.9 | 13.4 | 17.9 | Animals - Total number of animals named in 60 seconds  0-77  95=Physical problem  96=Cognitive/behavior problem  97=Other problem  98=Verbal refusal  -4 = Not available: UDS form submitted did not collect data in this way, or a skip pattern precludes response to this question |
| UDSBENTD | 0.0 | 17.0 | 5.2 | 3.7 | 8.3 | Total score for 10 to 15 minute delayed drawing of benson figure  0–17  95=Physical problem  96=Cognitive/behavior problem  97=Other problem  98=Verbal refusal  -4=Not available: UDS form submitted did not collect data in this way, or a skip pattern precludes response to this question |

**d:** MCI2AD_2.

| **Features Selected for MCI2AD_2** | **In total sample** | | | **In the sample who developed AD** | **In the sample who retained MCI** | **Descriptions** |
| --- | --- | --- | --- | --- | --- | --- |
|  | **Proportion in each response** | | | | |  |
| MEMORY | 0.0: 0.06  0.5: 0.56  1.0: 0.37  2.0: 0.01  3.0: 0.00 | | | 0.0: 0.01  0.5: 0.26  1.0: 0.71  2.0: 0.01  3.0: 0.00 | 0.0: 0.08  0.5: 0.74  1.0: 0.18  2.0: 0.00  3.0: 0.00 | Memory  0.0 = No impairment  0.5 = Questionable impairment  1.0 = Mild impairment  2.0 = Moderate impairment  3.0 = Severe impairment |
| COGORI | 0: 0.65  1: 0.35  9: 0.00 | | | 0: 0.34  1: 0.66  9: 0.00 | 0: 0.83  1: 0.17  9: 0.00 | Indicate whether the subject currently is meaningfully impaired, relative to previously attained abilities, in orientation  0=No  1 = Yes  9 = Unknown  -4= Not available: UDS form submitted did not collect data in this way, or a skip pattern precludes response to this question |
| DEP | 0: 0.81  1: 0.19 | | | 0: 0.82  1: 0.18 | 0: 0.81  1: 0.19 | Presumptive etiologic diagnosis - Depression  0 = No (assumed assessed and found not present)  1 = Yes |
| INDEPEND | 1: 0.62  2: 0.34  3: 0.03  4: 0.00  9: 0.01 | | | 1: 0.29  2: 0.61  3: 0.07  4: 0.00  9: 0.03 | 1: 0.80  2: 0.18  3: 0.01  4: 0.00  9: 0.00 | Level of independence  1=Able to live independently  2=Requires some assistance with complex activities  3=Requires some assistance with basic  activities  4=Completely dependent  9 = Unknown |
| JUDGMENT | 0.0: 0.32  0.5: 0.51  1.0: 0.17  2.0: 0.00 | | | 0.0: 0.07  0.5: 0.57  1.0: 0.35  2.0: 0.00 | 0.0: 0.46  0.5: 0.47  1.0: 0.07  2.0: 0.00 | Judgment and problem-solving  0.0 = No impairment  0.5 = Questionable impairment  1.0 = Mild impairment  2.0 = Moderate impairment  3.0 = Severe impairment |
| TOBAC30 | -4: 0.09  0: 0.87  1: 0.03  9: 0.01 | | | -4: 0.25  0: 0.72  1: 0.03  9: 0.01 | -4: 0.00  0: 0.96  1: 0.03  9: 0.01 | Smoked cigarettes in last 30 days  0=No  1=Yes  9 = Unknown  -4= Not available: UDS form submitted did not collect data in this way, or a skip pattern precludes response to this question |
| COGJUDG | 0: 0.43  1: 0.57  9: 0.00 | | | 0: 0.14  1: 0.86  9: 0.00 | 0: 0.59  1: 0.40  9: 0.00 | Indicate whether the subject currently is meaningfully impaired, relative to previously attained abilities, in executive function - judgment, planning, or problem-solving  0=No  1=Yes  9 = Unknown |
| COGSTAT | 0: 0.16  1: 0.00  2: 0.06  3: 0.30  4: 0.47 | | | 0: 0.17  1: 0.00  2: 0.01  3: 0.11  4: 0.71 | 0: 0.16  1: 0.01  2: 0.09  3: 0.41  4: 0.34 | Per clinician, based on the neuropsychological examination, the subject’s cognitive status is deemed  0=Clinician unable to render opinion 1=better than normal for age 2=Normal for age  3 = One or two test scores abnormal 4=Three or more scores are abnormal or lower than expected  9 = Missing  -4=Not available: UDS form submitted did not collect data in this way, or a skip pattern precludes response to this question |
| DECIN | 0: 0.11  1: 0.87  8: 0.02 | | | 0: 0.02  1: 0.98  8: 0.01 | 0: 0.17  1: 0.80  8: 0.03 | Does the co-participant report a decline in subject’s memory (relative to previously attained abilities)?  0=No  1=Yes  8=There is no co-participant  9 = Unknown |
| COGVIS | 0: 0.78  1: 0.21  9: 0.01 | | | 0: 0.66  1: 0.32  9: 0.02 | 0: 0.85  1: 0.14  9: 0.01 | Indicate whether the subject currently is meaningfully impaired, relative to previously attained abilities, in visuospatial function  0=No  1=Yes  9 = Unknown |
| FDGAD | 0: 0.04  1: 0.08  8: 0.87 | | | 0: 0.02  1: 0.16  8: 0.82 | 0: 0.06  1: 0.04  8: 0.90 | FDG-PET pattern of AD  0 = No  1 = Yes  8 = Unknown/not assessed  -4 = Not applicable: UDS form submitted did not collect data in this way, or a skip pattern precludes response to this question |
| AMYLPET | 0: 0.07  1: 0.14  8: 0.78 | | | 0: 0.01  1: 0.21  8: 0.78 | 0: 0.11  1: 0.11  8: 0.79 | Abnormally elevated amyloid on PET  0 = No  1 = Yes  8 = Unknown/not assessed  -4 = Not applicable: UDS form submitted did not collect data in this way, or a skip pattern precludes response to this question |
| COURSE | 1: 0.86  2: 0.01  3: 0.05  4: 0.01  5: 0.01  8: 0.05  9: 0.03 | | | 1: 0.97  2: 0.00  3: 0.01  4: 0.00  5: 0.00  8: 0.00  9: 0.01 | 1: 0.79  2: 0.01  3: 0.07  4: 0.01  5: 0.01  8: 0.07  9: 0.04 | Overall course of decline of cognitive/ behavioral/motor syndrome  1=Gradually progressive  2 = Stepwise  3 = Static  4 = Fluctuating  5 = Improved  8=Not applicable  9 = Unknown |
| MEDSIF | 1: 0.00  2: 0.01  7: 0.94  8: 0.05 | | | 1: 0.00  2: 0.00  7: 1.00  8: 0.00 | 1: 0.01  2: 0.01  7: 0.91  8: 0.08 | Primary, contributing, or non-contributing cause of cognitive impairment - medications  1 = Primary  2 = Contributing  3 = Non-contributing  7 = Cognitively impaired but no diagnosis of impairment due to medications  8 = Diagnosis of normal cognition  -4 = Not applicable: UDS form submitted did not collect data in this way, or a skip pattern precludes response to this question |
| AMYLCSF | 0: 0.03  1: 0.04  8: 0.93 | | | 0: 0.01  1: 0.08  8: 0.91 | 0: 0.04  1: 0.02  8: 0.94 | Abnormally low amyloid in CSF  0 = No  1 = Yes  8 = Unknown/not assessed  -4 = Not applicable: UDS form submitted did not collect data in this way, or a skip pattern precludes response to this question |
| HIPPATR | 0: 0.26  1: 0.30  8: 0.44 | | | 0: 0.16  1: 0.37  8: 0.46 | 0: 0.31  1: 0.26  8: 0.43 | Hippocampal atrophy  0 = No  1 = Yes  8 = Unknown/not assessed  -4 = Not applicable: UDS form submitted did not collect data in this way, or a skip pattern precludes response to this question |
| NACCNE4S | 0: 0.42  1: 0.38  2: 0.12  9: 0.09 | | | 0: 0.31  1: 0.42  2: 0.17  9: 0.11 | 0: 0.48  1: 0.35  2: 0.09  9: 0.08 | Number of APOE e4 alleles  0 = no e4 allele  1 = 1 copy of e4 allele  2 = 2 copies of e4 allele  9 = missing/unknown/not assessed |
| HYPOSOM | 0: 0.93  1: 0.07 | | | 0: 0.88  1: 0.12 | 0: 0.96  1: 0.04 | Hyposomnia/insomnia present  0 = No  1 = Yes  8 = Not assessed  -4 = Not available: UDS form submitted did not collect data in this way, or a skip pattern precludes response to this question |
| **Continuous Variables** | **In total sample** | | | **In sample who developed AD** | **In sample who retained as MCI** | **Descriptions** |
|  | **Min. value** | **Max. value** | **Mean** | **Mean** | **Mean** |  |
| UDSBENTD | 0.0 | 17.0 | 6.4 | 3.4 | 8.1 | Total score for 10 to 15 minute delayed drawing of benson figure  0–17  95=Physical problem 96=Cognitive/behavior problem 97=Other problem  98=Verbal refusal  -4=Not available: UDS form submitted did not collect data in this way, or a skip pattern precludes response to this question |
| TRAILA | 13.0 | 150.0 | 47.1 | 58.4 | 41.0 | Trail Making Test Part A - Total number of seconds to complete  0 - 150  995=Physical problem 996=Cognitive/behavior problem 997=Other problem  998=Verbal refusal  -4=Not available: UDS form submitted did not collect data in this way, or a skip pattern precludes response to this question |
